# Clinical and Parasitological Profiles of Gestational, Placental and Congenital Malaria in Northwestern Colombia

**DOI:** 10.3390/tropicalmed8060292

**Published:** 2023-05-25

**Authors:** Jaiberth Antonio Cardona-Arias, Luis Felipe Higuita-Gutiérrez, Jaime Carmona-Fonseca

**Affiliations:** 1 School of Microbiology, Universidad de Antioquia (UdeA), Medellín 050010, Colombia; hgfelipe87@gmail.com; 2 School of Medicine, Universidad Cooperativa de Colombia, Medellín 050012, Colombia; 3 Grupo de Investigación Salud y Comunidad César Uribe Piedrahíta, Universidad de Antioquia, Medellín 050010, Colombia; jaimecarmonaf@hotmail.com

**Keywords:** malaria, pregnancy, placenta, neonate, clinical symptoms, *Plasmodium*

## Abstract

This study compared the clinical–parasitological profiles of gestational (GM), placental (PM), and congenital (CM) malaria in northwestern Colombia. A cross-sectional study with 829 pregnant women, 549 placentas, and 547 newborns was conducted. The frequency of GM was 35.8%, PM 20.9%, and CM 8.5%. *P. vivax* predominated in GM; in PM, the proportion of *P. vivax* and *P. falciparum* was similar; in CM, *P. falciparum* predominated. The main clinical findings were headache (49%), anemia (32%), fever (24%), and musculoskeletal pain (13%). The clinical manifestations were statistically higher in *P. vivax* infections. In submicroscopic GM (positive with qPCR and negative with thick blood smear), the frequency of anemia, sore throat, and a headache was statistically higher compared with pregnant women without malaria. GM, PM, and CM reduce birth weight and head circumference. In Colombia, this is the first research on the clinical characteristics of GM, PM, and CM; contrary to evidence from other countries, *P. vivax* and submicroscopic infections are associated with clinical outcomes.

## 1. Introduction

World Health Organization (WHO) reported 247 million cases of malaria and 625,000 deaths in 2021 [1] without specific data on pregnancy-associated malaria (PAM). PAM includes three interrelated events: gestational malaria (GM) or infection by *Plasmodium* spp. in maternal peripheral blood, placental malaria (PM) or the presence of the parasite or its pigment hemozoin in this organ or in placental blood, and congenital malaria (CM), which corresponds to malaria of the newborn during the first week of life or later if there is no possibility of infection through a mosquito bite [2,3,4,5].

According to WHO experts, due to the low immune response during pregnancy, PAM is associated with a high risk of anemia and maternal death, abortion, stillbirth, premature delivery, low birth weight, and infant death. According to Centers for Disease Control and Prevention (CDC), the clinical profile of PAM varies by endemic level; thus, in regions of high transmission, the immune response prevents the progression of the infection, and asymptomatic cases predominate; however, in areas of low transmission, the immune response is not developed, which increases the probability of symptomatic and severe infections [6,7].

These clinical outcomes have been reported mainly in contexts with a predominance of *P. falciparum*, ignoring the specific clinical profile of *P. vivax* generally related to asymptomatic infections. Additionally, outside the African context, the role of *P. vivax* in PM and CM is poorly known, which supports the need for developing studies on clinical and parasitological profiles of the three forms of PAM in each context, with the aim of adjusting the intervention strategies to these profiles.

In Colombia, there are few clinical studies on PAM. Some epidemiological antecedents reported a high frequency of GM (35.8%), PM (27.7%), and CM (12.2%), associated with a higher risk of maternal anemia and lower birth weight [8,9,10]. In clinical practice guidelines for malaria, the clinical aspects of PAM are cited from WHO and CDC reports [11]. The Colombian guide does not cite investigations carried out in this country, where epidemiological surveillance focuses on clinical cases detected with thick blood smear (TBS); therefore, the infection profile based on PCR is unknown, and the few studies carried out register a high frequency of submicroscopic cases (positive with PCR and negative with TBS) [10].

There are few studies on PAM in Colombia without studies focused on its clinical aspects. Evidence of this is found in the following search strategies: “malaria pregnant*”[Title/Abstract] AND “clinical manifestations”[Title/Abstract] and “malaria pregnant*”[Title/Abstract] AND “clinical profile”[Title/Abstract], with which only one Colombian study was found, this research concluded that most cases in pregnant women correspond to uncomplicated infections [12], the other studies obtained correspond to other countries where anemia and severe malaria are highlighted as the main clinical outcomes of PAM [13,14]. Additionally, in Colombia, the clinical role of submicroscopic infections (in some cases are assumed as asymptomatic) and those caused by *P. vivax* (species to which lower pathogenicity is attributed) are not known.

Knowing the clinical–parasitological profile of PAM in the main endemic area of Colombia is important because of the following: (i) It generates evidence in an unexplored topic of malaria in the country. (ii) Clinical evidence is focused on countries with a predominance of *P. falciparum*; therefore, the main manifestations of *P. vivax* and its risk of causing PM and CM are unknown. (iii) Diagnosis is based on TBS, which generates underreporting and leads to ignorance of the clinical and epidemiological importance of submicroscopic infections. (iv) Surveillance of PAM in Colombia is limited to clinical cases, and paradoxically there are no clinical profiles for this disease. (v) In the search for PAM, only febrile symptomatic cases are screened, so this manuscript would help to expand the number of useful signs and symptoms to guide the search of patients or probable cases. (vi) Clinical–parasitological profile on PAM can help physicians to guide an early diagnosis, opportune and species-specific treatment, and avoid the consequences of PAM. The objective of this research was to compare the clinical–parasitological profile of GM, PM, and CM malaria, in northwestern Colombia. The specific objectives were as follows: (i) compare the frequency of clinical findings in each one of the three forms of PAM (gestational, placental, and congenital) comparing positive GM, PM, and CM versus negative pregnant women; (ii) compare the clinical findings between *P. vivax*, *P. falciparum*, mixed-malaria infections in GM, PM, and CM; (iii) compare the frequency of clinical findings in microscopic GM, PM, and CM versus the submicroscopic infections and negative pregnant women.

## 2. Materials and Methods

### 2.1. Type and Subjects of Study

Cross-sectional in three different analysis units: for GM, we included 829 pregnant women; for PM, 549 placentas were included; and for CM, 547 newborns were included (379 with umbilical cord blood, 233 peripheral blood). Pregnant women were recruited in three local hospitals in the main malaria-endemic area of Colombia, located in the northwest of the country. This region reports approximately 60% of annual malaria cases in Colombia; it is highly endemic with an annual parasite index >25/1000 exposed, transmission is stable throughout the year, and *P. vivax* predominates (60–70% of cases), but *P. falciparum* also circulates [15].

**Figure 1:**
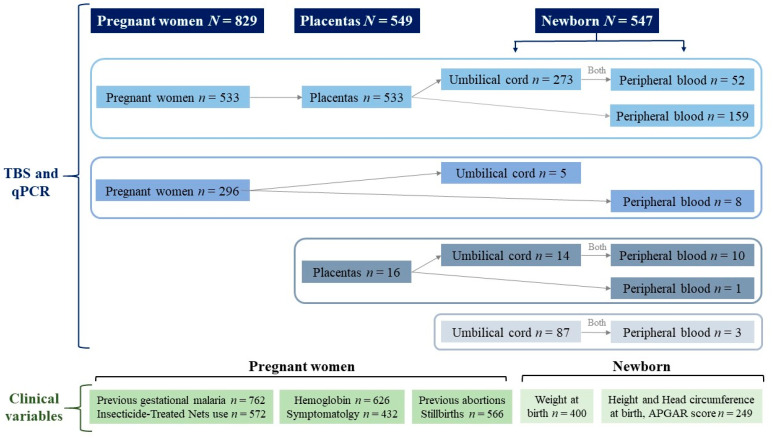
Flowchart of the recruitment process.

It was not possible to have the same number of pregnant women, placentas, and newborns for multiple economic, social, or health system factors, such as the following examples: a pregnant woman was included, and we could not obtain the placenta or newborns sample because she presented a complication or risk for which her delivery was attended in another health institution (in the capital of the Department from Córdoba-Colombia); some only consented to take one of the samples (for example blood from the pregnant woman or from the cord); others entered during pregnancy but did not sign (or withdrew) the consent for the placenta or newborn samples; the team that attended the delivery or the family of the pregnant women did not contact the research group to notify them of the date of delivery, among other economic and cultural reasons that prevented the delivery care at the local hospital (Figure 1).

Pregnant women and newborns met the following inclusion criteria: receiving antenatal care or delivery at the local hospital of their municipality, pregnant women residing in the study region for at least one year, and having TBS and qPCR results for malaria. Pregnant women and newborns diagnosed with other diseases, infections, or complications of their health status, on antimalarial treatment at the time of the study, or without signing informed consent were excluded. The samples were collected between 2010 and 2019 using the same standardized procedures for collecting information and diagnosing malaria, which consist of applying the same form for extracting variables from the medical chart and using the same survey. For the processing of this information, the research group trained and standardized three nursing assistants who led the information collection.

### 2.2. Diagnosis of Malaria Infection

TBS and qPCR were performed on the same blood samples (at the same time). The diagnosis of GM was made with peripheral blood collected by venipuncture (pregnant women were recruited in trimesters 2–3 of pregnancy, both symptomatic and asymptomatic). In PM, placental blood was taken from a point near the insertion of the umbilical cord and another in the distal part of this organ; in CM, peripheral blood from the newborn by heel puncture or umbilical cord was used. In the case of a pregnant woman or newborn positive for TBS, antimalarial treatment was performed in accordance with the Colombian clinical practice guideline. In submicroscopic cases, we notified the physician of the result of the qPCR, and depending on his clinical evaluation, the doctor decided on the treatment.

For qPCR, Whatmann #3 filter paper was used for DNA extraction with the Saponin-Chelex method. PCR is the main molecular diagnosis of malaria with a detection limit of <0.02 parasites/μL. qPCR avoids post-amplification manipulation and quantifies the number of microorganisms; it includes a highly conserved region 18S rRNA of *Plasmodium* and primers polymorphic specific to *P. falciparum*, *P. vivax*, *P. malariae*, and *P. ovale* [16]. TBS is considered positive when a minimum of 200 microscopic fields are observed under 100× magnification and at least one parasitic form is found. Based on this, the presence of *P. falciparum*, *P. vivax*, or mixed malaria is defined. The latter corresponds to the observation of parasitic forms of *P. vivax* and concomitant gametocytes of *P. falciparum* or regular asexual forms compatible with *P. falciparum* in a proportion of ≥40% of the 100 parasitic forms observed [17].

### 2.3. Source of Clinical Information

The following variables were extracted from the medical chart of pregnant women: hemoglobin (values less than 11.0 g/dL were considered anemia) [18], presence or absence of headache, fever, musculoskeletal, abdominal and throat pain, chills, vomiting, cough, sweating, diarrhea, asthenia, adynamia, choluria, loss of appetite, nausea, conjunctival and palmar pallor, previous abortions, and stillbirths. Extracted data from the newborn were weight, height, head circumference at birth, and the APGAR score (a newborn test that measures respiratory effort, heart rate, muscle tone, reflexes, and skin color, minutes 1 and 5). When taking the blood samples, they were asked about the previous diagnosis of malaria in the current pregnancy and insecticide-treated bed net use. In the variables, the sample sizes were heterogeneous (there are missing values), given that, in many cases, the medical chart does not record these variables exhaustively (Figure 1).

### 2.4. Statistical Analysis

Categorical variables were described with absolute (#) and relative (%) frequencies, and the continuous variables were described with median and interquartile range (not present a normal distribution according to the Kolmogorov–Smirnov with Liliefors correction). The comparison of the clinical findings between positive and negative for PAM and those affected by *P. falciparum*, *P. vivax,* or mixed malaria was performed with the Pearson chi-square test or Fisher’s exact test (n < 5). When the comparison of *P. falciparum*, *P. vivax,* or mixed malaria showed significant *p* values, multiple comparisons were made with the Bonferroni statistic. In order to compare the continuous variables with PM and CM, the Mann–Whitney U test was used; for causal species and microscopic or submicroscopic GM, the Kruskal–Wallis H with post hoc of Dunnett was used. A *p*-value < 0.05 was considered statistically significant.

### 2.5. Ethic

The Declaration of Helsinki and the specifications of Resolution 8430 of Colombia for research with pregnant women were applied. All pregnant women signed the informed consent (of legal age) or assent (under 18 years of age) obtained in writing with the signature of the pregnant woman and a witness external to the research group. This study was classified as minimal risk, endorsed by the Ethics Committee of the University Research Headquarters (SIU), University of Antioquia, Act 21-101-961 of the year 2021. All data were anonymized to guarantee confidentiality.

## 3. Results

The frequency of GM was 35.8% (297/829). Among the 297 positives, 45.1% were submicroscopic, 63% were *P. vivax*, 34.3% were *P. falciparum*, and 2.7% were mixed malaria. The frequency of PM was 20.9% (115/549), with 94.8% submicroscopic, an equal proportion of *P. vivax* and *P. falciparum* (44.3%), and 11.3% mixed malaria. CM was 12.4% in umbilical cord blood and 2.2% in peripheral blood of the newborn, all submicroscopic cases, and with *P. falciparum* as the main causal species with 63.5% of the cases (Table 1). The concordance between qPCR and TBS in GM was 83%; in PM, it was 76%; and in CM, it was 92%, mainly explained by the concordance in the negative results. In 432 subjects in whom it was possible to obtain samples, the pregnant woman, the placenta, and the newborn (Figure 1), 24 (5.6%) were found positive for the three events (GM, PM, and CM). In the subgroup of 123 positive pregnant women (GM) from whom the placental sample was available, the frequency of PM was 51.2% (n = 63), which was probably reinfection since these pregnant women were included during the first or second trimester of pregnancy and received treatment.

In pregnant women, the most frequent signs and symptoms were headache (48.6%), fever (23.8%), musculoskeletal pain (13.0%), shaking chills (10.9%), and vomiting (10.9%). Previous abortions occurred in 15.4% (87/566) and stillbirths in 2.1% (12/566). The following medians (interquartile range) were presented in the newborn: birth weight 3100 g (2852–3400), height 50 cm (49–51), head circumference 34 cm (33–34), and APGAR score 8 (7–9).

We did not find an association between GM and diarrhea, but with all other clinical manifestations, there was a statistical association. According to the causal species, clinical findings were more frequent in women with *P. vivax*, except for sore throat and cough (Table 2). There was no statistical association of GM with abortions (*p* Chi^2^ = 0.293), stillbirths (*p* Chi^2^ = 0.342), or ITN use (*p* Chi^2^ = 0.086).

In submicroscopic GM, a statistically higher frequency of anemia, cough, sore throat, and headaches was found compared to negative pregnant women; the first three symptoms were statistically the same as those found in pregnant women with microscopic malaria (positive with both diagnostic tests) (Table 3). We did not find an association between submicroscopic GM and previous abortions (*p* Chi^2^ = 0.677) or stillbirths (*p* Chi^2^ = 0.498).

PM presented statistical differences with previous GM in the current pregnancy. In positives for PM, the frequency of the previous GM was 45.7% (vs. 22.0% in negative PM). There was no statistical association of PM with previous abortions (*p* Chi^2^ = 0.490) or stillbirths (*p* Chi^2^ = 0.193). PM presented statistical differences with anemia, and there was no significant association with the other clinical findings (Table 4). We did not find an association between CM and the mother’s clinical findings.

In the outcomes in the newborn, GM, PM, and CM did not present a statistical relationship with the APGAR score or with the height at birth; when disaggregating these comparisons according to the causal species, an association (Kruskal–Wallis *p* = 0.012) was only found between height and mixed GM. In the mixed-GM group, the median was 45 cm (IR 45–46), and in negatives and pregnant women with *P. falciparum* and *P. vivax,* the median was 50 (IR 49–51).

Weight and head circumference were statistically lower in newborns with a history of GM and PM and in those with CM. Submicroscopic GM was associated with lower birth weight and head circumference. Depending on the causal species, mixed GM and mixed CM were associated with lower birth weights compared with *P. falciparum* and *P. vivax* infections. CM by *P. falciparum* was associated with lower birth weight (Table 5). In the subgroup positive for the three events (GM, PM, and CM), only statistical differences were found in birth weight, with a median of 3.1 (IR = 2.9–3.4) within negatives and 2.9 (IR = 2.7–3.0) within positives.

## 4. Discussion

The frequency of GM was 35.8%, PM 20.9%, and CM 8.5%; this coincides with a meta-analysis of the studies from Colombia [19]. The high frequency of submicroscopic infection points to the need to use molecular diagnostics, given its association with lower hemoglobin levels, anemia, low birth weight, premature births, and perpetuation of the transmission of *Plasmodium* [20,21].

In GM, *P. vivax* prevailed; this is consistent with the higher frequency of this species in the study region [15], but the predominance of *P. falciparum* in CM could mean greater possibilities of crossing the placenta of *P. falciparum,* as has been described by other authors [22].

In Colombia, there are no studies on the clinical characteristics of PAM to compare our results, but a study with 112 patients of the general population diagnosed with TBS presented a different clinical profile: 99% with fever, 94% sweating, and 94% musculoskeletal pain. Most of the signs and symptoms (diarrhea, listlessness, palmar pallor, dry mucous membranes) were statistically more frequent in *P. falciparum*, and only chills it was more frequent in *P. vivax* [23]; this is different from our results in pregnant women, which demonstrate that pregnancy generates changes in the clinical profile.

Submicroscopic GM presented an association with anemia, cough, sore throat, and headache, showing the need to implement molecular diagnostics, given the false negatives of TBS. Other publications report the same situation but focus their arguments on the sensitivity problems of TBS in non-febrile patients, the need to interrupt their transmission or the achievement of the goals of pre-elimination or elimination [16,24,25,26]. Our research adds evidence to the importance of molecular diagnosis in Colombia because submicroscopic infections were associated with clinical outcomes similar to microscopic or symptomatic infections.

In addition, our results are relevant to support subsequent research on the clinical utility of PAM molecular diagnosis since some authors indicate that PCR does not provide information on the viability of the microorganism; therefore, its clinical use is inadequate (similar to rapid test based on PFHRP2 and other proteins that can produce false positives), so the search for symptomatic cases and the prescription of the treatment of malaria should continue to be carried out with TBS. In this sense, this study shows that submicroscopic cases presented some clinical findings statistically similar to those of microscopic PAM [27].

In PM, the frequency of anemia was higher in *P. vivax*. Although higher pathogenicity has been reported for *P. falciparum* [22], the evidence provided by this research is added to reviews that described the pathophysiology of *P. vivax* with serious effects such as anemia and other hematological complications, severe malaria, acute respiratory distress, and multiple organ failure [28], as well as Colombian studies that reported the pathological effects of *P. vivax* in PAM [8,9,10]. It is important to highlight that the frequency of GM, parasitemia density, and the infection trimester are the main risk factors for PM; therefore, intermittent preventive treatment, screening, and treatment of GM from the first trimester must be provided [29,30].

CM was associated with a reduction in weight and head circumference at birth. The effect on birth weight was higher in mixed malaria and *P. falciparum*. This coincides with the available evidence on this topic [6,7,10,19] and is worrying given that recent studies have reported a high frequency of low birth weight, preterm birth, and perinatal mortality in areas with stable malaria transmission, where intermittent preventive treatment in pregnancy with sulfadoxine–pyrimethamine has not been effective to reduce CM or low birth weight [31,32]. Additionally, in Colombia, CM has not been detected because the health system and the malaria control program are based on TBS when most cases are submicroscopic [19].

Regarding head circumference at birth, several studies have reported similar results, evidencing an association of PM with a decrease in head circumference; newborns with a small head in pregnant women infected with *P falciparum* or fetal and neonatal outcomes avoided when GM is treated during pregnancy [33,34,35,36].

### Limitations and Strengths of This Research

The way in which health care is structured in Colombia restricts the number of times pregnant women are attended by health personnel; thus, important data from the medical chart are frequently omitted. The foregoing resulted in many women in this investigation not having a complete record of clinical manifestations. Molecular diagnosis with PCR is excluded as part of the treatment guidelines, with the placenta as a waste organ in hospitals, which leads to difficulties for studies of PM and structural barriers to accessing antenatal care in dispersed rural areas that are most affected by malaria, leading to problems of recruitment of all pregnant women exposed or at risk.

## 5. Conclusions

This research is one of the few available in Colombia on the clinical characteristics of PAM. Contrary to evidence from other countries, this study showed that submicroscopic infections and those caused by *P. vivax* are associated with clinical findings. The differences in the causal species of GM, PM, and CM evidence the complexity of the parasitological component of PAM. The need to improve the completion of the medical chart and increase the number of studies on medical aspects of gestational, placental, and congenital malaria was also demonstrated.

## Figures and Tables

**Table 1:** Frequency of gestational, placental, and congenital plasmodial infection, and causal species.

	Plasmodial Infection According to qPCR and Thick Blood Smear			
	Gestational	Placental	Congenital	
			Cord	Newborn
The number of samples	829	549	379	233
Positive for *Plasmodium* % (*n*)	35.8 (297/829)	20.9 (115/549)	12.4 (47/379)	2.2 (5/233)
Submicroscopic (positive PCR and negative TBS)				
% (*n*) of total	16.2 (134/829)	19.9 (109/549)	12.4 (47/379)	2.2 (5/233)
% (*n*) of positives	45.1 (134/297)	94.8 (109/115)	100 (47/47)	100 (5/5)
*P. vivax*				
% (*n*) of total	22.6 (187/829)	9.3 (51/549)	3.4 (13/379)	1.3 (3/233)
% (*n*) of positives	63.0 (187/297)	44.3 (51/115)	27.7 (13/47)	60.0 (3/5)
*P. falciparum*				
% (*n*) of total	12.3 (102/829)	9.3 (51/549)	8.2 (31/379)	0.9 (2/233)
% (*n*) of positives	34.3 (102/297)	44.3 (51/115)	65.9 (31/47)	40.0 (2/5)
Mixed-malaria				
% (*n*) of total	1.0 (8/829)	2.4 (13/549)	0.8 (3/379)	0
% (*n*) of positives	2.7 (8/297)	11.3 (13/115)	6.4 (3/47)	0

**Table 2:** Clinical symptoms and signs according to gestational plasmodial infection and causal species.

	Total N = 432 % (n)	Gestational Malaria		*p* Chi^2^	Causal Species		
		Negative N = 312	Positive N = 120		*P. falciparum* N = 24	*P. vivax* N = 94	*p* Chi^2^
		% (n) ^a^	% (n) ^a^		% (n) ^a^	% (n) ^a^	
Headache	48.6 (210)	39.1 (122)	73.3 (88)	<0.001	50.0 (12)	80.9 (76)	<0.001 ^b^
Fever	23.8 (103)	10.3 (32)	59.2 (71)	<0.001	8.3 (2)	73.4 (69)	<0.001 ^b^
Musculoskeletal ain	13.0 (56)	6.7 (21)	29.2 (35)	<0.001	4.2 (1)	36.2 (34)	<0.001 ^b^
Shaking chills	10.9 (47)	7.4 (23)	20.0 (24)	<0.001	4.2 (1)	24.5 (23)	<0.001 ^b^
Vomiting	10.9 (47)	8.0 (25)	18.3 (22)	0.002	0.0 (0)	23.4 (22)	<0.001 ^b^
Abdominal pain	7.4 (32)	5.8 (18)	11.7 (14)	0.036	4.2 (1)	13.8 (13)	0.060
Cough	4.9 (21)	3.2 (10)	9.2 (11)	0.010	16.7 (4)	7.4 (7)	0.014
Sweating	5.3 (23)	0.3 (1)	18.3 (22)	<0.001	0.0 (0)	23.4 (22)	<0.001 ^b^
Diarrhea	4.4 (19)	4.8 (15)	3.3 (4)	0.503	0.0 (0)	4.3 (4)	0.723
Asthenia–Adynamia	4.6 (20)	0.0 (0)	16.7 (20)	<0.001	0.0 (0)	21.3 (20)	<0.001 ^b^
Coluria	3.9 (17)	2.2 (7)	8.3 (10)	0.004	4.2 (1)	9.6 (9)	0.016 ^b^
Loss of appetite	4.2 (18)	0.0 (0)	15.0 (18)	<0.001	0.0 (0)	19.1 (18)	<0.001 ^b^
Nausea	3.2 (14)	0.0 (0)	11.7 (14)	<0.001	0.0 (0)	14.9 (14)	<0.001 ^b^
Sore throat	2.3 (10)	0.6 (2)	6.7 (8)	<0.001	8.3 (2)	6.4 (6)	0.002
Conjunctival Pallor	2.5 (11)	0.0 (0)	9.2 (11)	<0.001	0.0 (0)	11.7 (11)	<0.001 ^b^
Palmar pallor	1.9 (8)	0.0 (0)	6.7 (8)	<0.001	0.0 (0)	8.5 (8)	<0.001 ^b^
Maternal Anemia ^c^	32.3 (202/626)	24.9 (103/414)	46.7 (99/212)	<0.001	35.8 (29/81)	54.8 (69/126)	<0.001 ^b^

^a^ In percentages the denominator is N in each column, and the numerator is the pregnant women who register each clinical finding. ^b^ Post hoc showed that the group with *P. vivax* was statistically higher. ^c^ In this variable, “n” is different from that reported in the other clinical findings (it does not include a case of mixed-GM).

**Table 3:** Clinical findings in microscopic and submicroscopic gestational malaria.

	Without Gestational Malaria (A) N = 312 % (n)	Submicroscopic Gestational Malaria (B) N = 38 % (n)	Microscopic Gestational Malaria (C) N = 82 % (n)	*p* (X^2^)
Bonferroni C = B > A				
Maternal anemia ^a^	24.9 (103/414)	45.7 (59/129)	47.0 (39/83)	<0.001
Cough	3.2 (10)	10.5 (4)	8.5 (7)	0.032
Sore throat	0.6 (2)	5.3 (2)	7.3 (6)	0.001
Bonferroni C > B > A				
Headache	39.1 (122)	47.4 (18)	85.4 (70)	<0.001
Bonferroni C > A = B				
Fever	10.3 (32)	7.9 (3)	82.9 (68)	<0.001
Coluria	2.2 (7)	2.6 (1)	11.0 (9)	0.001
Bonferroni C > A > B				
Shaking chills	7.4 (23)	2.6 (1)	28.0 (23)	<0.001
Musculoskeletal pain	6.7 (21)	0.0 (0)	42.7 (35)	<0.001
Vomiting	8.0 (25)	0.0 (0)	26.8 (22)	<0.001
Abdominal pain	5.8 (18)	0.0 (0)	17.1 (14)	<0.001
Sweating	0.3 (1)	0.0 (0)	26.8 (22)	<0.001
Asthenia–adynamia	0.0 (0)	0.0 (0)	24.4 (20)	<0.001
The lack of appetite	0.0 (0)	0.0 (0)	22.0 (18)	<0.001
Nausea	0.0 (0)	0.0 (0)	17.1 (14)	<0.001
Conjunctival pallor	0.0 (0)	0.0 (0)	13.4 (11)	<0.001
Palmar pallor	0.0 (0)	0.0 (0)	9.8 (8)	<0.001

In percentages, the denominator is N in each column, and the numerator is the pregnant women who register each clinical finding. ^a^ In this variable, “n” is different from that reported in the other clinical findings of the table.

**Table 4:** Clinical symptoms and signs according to placental plasmodial infection and causal species.

	Placental Malaria		*p* Fisher	Causal Species		
	Negative N = 396	Positive N = 36		*P. falciparum* N = 14	*P. vivax* N = 17	*p* Chi^2^
	% (n) ^a^	% (n) ^a^		% (n) ^a^	% (n) ^a^	
Headache	45.2 (179)	47.2 (17)	0.816 ^b^	57.1 (8)	47.1 (8) ^c^	0.529
Fever	14.9 (59)	16.7 (6)	0.777 ^b^	14.3 (2)	23.5 (4)	0.603
Musculoskeletal pain	7.1 (28)	11.1 (4)	0.327	14.3 (2)	11.8 (2)	0.593
Shaking chills	9.1 (36)	16.7 (6))	0.142 ^b^	14.3 (2)	21.4 (3)	0.515
Vomiting	7.8 (31)	2.8 (1)	0.501	0 (0)	5.9 (1)	0.636
Abdominal pain	3.0 (12)	0 (0)	0.609	0 (0)	0 (0)	0.772
Cough	6.1 (24)	0 (0)	0.246	0 (0)	0 (0)	0.511
Sweating	0 (0)	0 (0)	--	0 (0)	0 (0)	--
Diarrhea	3.0 (12)	0 (0)	0.609	0 (0)	0 (0)	0.772
Asthenia–adynamia	0 (0)	0 (0)	--	0 (0)	0 (0)	--
Coluria	3.8 (15)	0 (0)	0.625	0 (0)	0 (0)	0.703
Loss of appetite	0 (0)	0 (0)	--	0 (0)	0 (0)	--
Nausea	0 (0)	0 (0)	--	0 (0)	0 (0)	--
Sore throat	2.3 (9)	0 (0)	1.000	0 (0)	0 (0)	0.841
Conjunctival pallor	0 (0)	0 (0)	--	0 (0)	0 (0)	--
Palmar pallor	0 (0)	0 (0)	--	0 (0)	0 (0)	--
Anemia ^d^	22.0 (62/281)	45.7 (43/94)	<0.001	48.9 (23/47)	54.3 (19/35)	<0.001

^a^ In percentages, the denominator is N in each column, and the numerator is the pregnant women who register each clinical finding. ^b^ Chi^2^. ^c^ Five cases of PM-mixed were negative for signs and symptoms, and only one presented a headache. ^d^ In this variable, “n” is different from that reported in the other clinical findings of the table.

**Table 5:** Weight and head circumference at birth according to the type of malaria and causal species.

	Weight Kg s		Head Circumference cm		Height at Birth cm	APGAR Score
	n	Median (IR)	n	Median (IR)	Median (IR) ^a^	Median (IR) ^a^
Gestational Malaria						
Negative	307	3.2 (2.9–3.4)	216	34 (33–35)	50 (49–51)	8 (7–9)
Microscopic	20	3.0 (2.7–3.2) ^b^	8	34 (32–35)	50 (49–50)	8 (7–9)
Submicroscopic	73	3.1 (2.7–3.2) ^b^	25	33 (32–34) ^b^	49 (48–51)	8 (8–9)
p Kruskal–Wallis		0.027		0.031	0.654	0.694
Causal species of gestational malaria						
Negative	307	3.2 (2.9–3.4)	216	34 (33–35)	50 (49–51)	8 (7–9)
*P. vivax*	45	3.0 (2.8–3.3) ^b^	15	33 (32–34) ^b^	50 (49–50)	8 (8–9)
*P. falciparum*	44	2.9 (2.7–3.1) ^b^	16	34 (33–34)	49 (48–51)	8 (7–9)
Mixed	4	2.5 (2.3–2.7) ^b c^	2	31 (31–31) ^b c^	45 (45–46) ^b c^	9 (9–9)
P Kruskal–Wallis		0.001		0.001	0.012	0.332
Placental Malaria						
Negative	296	3.2 (3.0–3.5)	223	34 (33–35)	50 (49–51)	8 (7–9)
Positive	104	2.9 (2.6–3.1)	26	33 (32–34)	50 (48–51)	8 (7–9)
p Mann–Whitney		<0.001		0.001	0.216	0.728
Causal species of placental malaria						
Negative	296	3.2 (2.9–3.5)	223	34 (33–35)	50 (49–51)	8 (7–9)
*P. vivax*	46	2.9 (2.6–3.0) ^b^	17	33 (32–34) ^b^	50 (48–50)	8 (7–9)
*P. falciparum*	47	2.9 (2.6–3.1) ^b^	7	34 (32–34) ^b^	50 (48–51)	8 (7–9)
Mixed	11	2.9 (2.2–3.2) ^b^	2	32 (32–32) ^b^	50 (50–51)	9 (8–9)
p H de Kruskal–Wallis		<0.001		0.007	0.385	0.822
Congenital Malaria						
Negative	350	3.1 (2.9–3.4)	240	34 (33–34)	50 (49–51)	8 (7–9)
Positive	50	3.0 (2.7–3.2)	9	32 (32–33)	49 (49–50)	8 (7–9)
p Mann–Whitney		0.007		0.013	0.518	0.756
Causal species of congenital malaria						
Negative	350	3.1 (2.9–3.4)	240	34 (33–34)	50 (49–51)	8 (7–9)
*P. vivax*	16	3.0 (2.9–3.1) ^b^	3	32 (32–34) ^b^	50 (49–50)	8 (7–8)
*P. falciparum*	32	2.9 (2.7–3.2) ^b d^	6	33 (33–33) ^b^	49 (49–49)	9 (9–9)
Mixed	2	2.7 (2.5–8.8) ^b c^	--	--	--	--
p Kruskal–Wallis		0.043		0.038	0.748	0.453

^a^ The “n” of each row is the same as that reported in the head circumference. ^b^ Post hoc test showed statistical differences compared to the negatives. ^c^ Post hoc test showed statistical differences against *P. falciparum* and *P. vivax*. ^d^ Post hoc test showed statistical differences against *P. vivax*.

## Data Availability

All relevant data are cited in the manuscript.

## References

[1] World Health Organization World Malaria Report 2022. https://www.who.int/teams/global-malaria-programme/reports/world-malaria-report-2022.

[2] Agenjo González M. (2014). Malaria and Pregnancy: Complications, Prevention and Treatment.

[3] Liu Y., Griffin J.B., Muehlenbachs A., Rogerson S.J., Bailis A.J., Sharma R., Sullivan D.J., Tshefu A.K., Landis S.H., Kabongo J.-M.M. (2016). Diagnosis of placental malaria in poorly fixed and processed placental tissue. Malar. J..

[4] Agudelo-García O.M., Arango-Flórez E.M., Carona-Fonseca J. (2017). Submicroscopic and asymptomatic congenital infection by *Plasmodium vivax* or *P. falciparum* in Colombia: 37 Cases with placental histopathology and cytokine profile in maternal and placental blood. J. Trop. Med..

[5] Menendez C., Mayor A. (2007). Congenital malaria: The least known consequence of malaria in pregnancy. Semin. Fetal Neonatal. Med..

[6] World Health Organization, Malaria Policy Advisory Group—WHO (2017). Meeting Report of the Evidence Review Group on Malaria in Pregnancy.

[7] CDC Intermittent Preventive Treatment of Malaria for Pregnant Women (IPTp). 23 July 2018. https://www.cdc.gov/malaria/malaria_worldwide/reduction/iptp.html#:~:text=Malaria%20infection%20during%20pregnancy%20can,a%20risk%20factor%20for%20death.

[8] Cardona-Arias J.A., Carmona-Fonseca J. (2022). Frequency of gestational malaria and maternal–neonatal outcomes, in Northwestern Colombia 2009–2020. Sci. Rep..

[9] Cardona-Arias J.A., Carmona-Fonseca J. (2022). Frequency of placental malaria and its associated factors in northwestern Colombia, pooled analysis 2009–2020. PLoS ONE.

[10] Cardona-Arias J.A., Carmona-Fonseca J. (2022). Congenital malaria: Frequency and epidemiology in Colombia, 2009–2020. PLoS ONE.

[11] Ministerio de Salud y Protección Social de Colombia (2020). Guía de Práctica Clínica Diagnóstico y Tratamiento de la Malaria.

[12] Lopez-Perez M., Pacheco M.A., Buriticá L., Escalante A.A., Herrera S., Arévalo-Herrera M. (2016). Malaria in pregnancy: A passive surveillance study of pregnant women in low transmission areas of Colombia, Latin America. Malar. J..

[13] Assabri A.M., Muharram A.A. (2002). Malaria in pregnancy in Hodiedah, Republic of Yemen. East Mediterr. Health J..

[14] Kwizera A., Ntasumumuyange D., Small M., Rulisa S., Moscovitz A.N., Magriples U. (2021). Assessment of perinatal outcomes of pregnant women with severe versus simple malaria. PLoS ONE.

[15] Carmona-Fonseca J. (2017). La Región “Urabá Antioqueño-Cuencas altas de los ríos Sinú y San Jorge-Bajo Cauca Antioqueño”: “Guarida” del paludismo colombiano Revista de la Universidad Industrial de Santander. Salud.

[16] Zheng Z., Cheng Z. (2017). Advances in Molecular Diagnosis of Malaria. Adv. Clin. Chem..

[17] Instituto Nacional de Salud de Colombia (2015). Manual para el Diagnóstico de Malaria no Complicada en Puestos de Diagnóstico y Tratamiento.

[18] World Health Organization (2011). Haemoglobin Concentrations for the Diagnosis of Anaemia and Assessment of Severity.

[19] Cardona-Arias J.A., Carmona-Fonseca J. (2021). Meta-analysis of the prevalence of malaria associated with pregnancy in Colombia 2000–2020. PLoS ONE.

[20] Cottrell G., Moussiliou A., Luty A.J., Cot M., Fievet N., Massougbodji A., Deloron P., Tuikue Ndam N. (2015). Submicroscopic *Plasmodium falciparum* infections are associated with maternal anemia, premature births, and low birth weight. Clin. Infect. Dis..

[21] Alkan M.L. (2020). The importance of submicroscopic diagnosis of malaria. Clin. Infect. Dis..

[22] Moxon C.A., Gibbins M.P., McGuinness D., Milner D.A., Marti M. (2020). New Insights into Malaria Pathogenesis. Annu. Rev. Pathol..

[23] Knudson A., Sánchez R., Pérez M., Cortés L., Guerra A., Nicholls R. (2015). Perfil clínico y parasitológico de la malaria por *Plasmodium falciparum* y *Plasmodium vivax* no complicada en Córdoba, Colombia. Rev. Fac. Med..

[24] Lin J.T., Saunders D.L., Meshnick S.R. (2014). The role of submicroscopic parasitemia in malaria transmission: What is the evidence?. Trends Parasitol..

[25] Chen I., Clarke S.E., Gosling R., Hamainza B., Killeen G., Magill A., O’Meara W., Price R.N., Riley E.M. (2016). “Asymptomatic” malaria: A chronic and debilitating ifection that should be treated. PLoS Med..

[26] Oyegoke O.O., Maharaj L., Akoniyon O.P., Kwoji I., Roux A.T., Adewumi T.S., Maharaj R., Oyebola B.T., Adeleke M.A., Okpeku M. (2022). Malaria diagnostic methods with the elimination goal in view. Parasitol. Res..

[27] Rantala A.M., Taylor S.M., Trottman P.A., Luntamo M., Mbewe B., Maleta K., Kulmala T., Ashorn P., Meshnick S.R. (2010). Comparison of real-time PCR and microscopy for malaria parasite detection in Malawian pregnant women. Malar. J..

[28] Dayananda K.K., Achur R.N., Gowda D.C. (2018). Epidemiology, drug resistance, and pathophysiology of *Plasmodium vivax* malaria. J. Vector Borne Dis..

[29] Briggs J., Ategeka J., Kajubi R., Ochieng T., Kakuru A., Ssemanda C., Wasswa R., Jagannathan P., Greenhouse B., Rodriguez-Barraquer I. (2019). Impact of microscopic and submicroscopic parasitemia during pregnancy on placental malaria in a high-transmission setting in Uganda. J. Infect. Dis..

[30] Zakama A.K., Ozarslan N., Gaw S.L. (2020). Placental Malaria. Curr. Trop. Med. Rep..

[31] Otuli Noël L., Nguma Jean-Didier B., Alongo Mike-Antoine M., Bosunga Gedeon K., Mukonkole Jean-Paulin M., Likwela Joris L., Okenge Jean-Pascal M. (2020). Prevalence of Congenital Malaria in Kisangani, A Stable Malaria Transmission Area in Democratic Republic of the Congo. Infect. Dis. Obstet. Gynecol..

[32] Fitri L.E., Jahja N.E., Huwae I.R., Nara M.B., Berens-Riha N. (2014). Congenital malaria in newborns selected for low birth-weight, anemia, and other possible symptoms in Maumere, Indonesia. Korean J. Parasitol..

[33] Meuris S., Piko B.B., Eerens P., Vanbellinghen A.M., Dramaix M., Hennart P. (1993). Gestational malaria: Assessment of its consequences on fetal growth. Am. J. Trop. Med. Hyg..

[34] Walther B., Miles D.J., Waight P., Palmero M.S., Ojuola O., Touray E.S., Whittle H., van der Sande M., Crozier S., Flanagan K.L. (2012). Placental malaria is associated with attenuated CD4 T-cell responses to tuberculin PPD 12 months after BCG vaccination. BMC Infect. Dis..

[35] Dombrowski J.G., Souza R.M., Lima F.A., Bandeira C.L., Murillo O., Costa D.S., Peixoto E.P.M., Cunha M.D.P., Zanotto P.M.A., Bevilacqua E. (2019). Association of Malaria Infection During Pregnancy With Head Circumference of Newborns in the Brazilian Amazon. JAMA Netw. Open.

[36] Koladjo B.F., Yovo E., Accrombessi M., Agbota G., Atade W., Ladikpo O.T., Mehoba M., Degbe A., Jackson N., Massougbodji A. (2022). Malaria in the First Trimester of Pregnancy and Fetal Growth: Results from a Beninese Preconceptional Cohort. J. Infect. Dis..

